# PrP^C^ knockdown by liposome-siRNA-peptide complexes (LSPCs) prolongs survival and normal behavior of prion-infected mice immunotolerant to treatment

**DOI:** 10.1371/journal.pone.0219995

**Published:** 2019-07-22

**Authors:** Heather Bender, Noelle Noyes, Jessica L. Annis, Amanda Hitpas, Luke Mollnow, Kendra Croak, Sarah Kane, Kaitlyn Wagner, Steven Dow, Mark Zabel

**Affiliations:** 1 Prion Research Center, Department of Microbiology, Immunology and Pathology, College of Veterinary Medicine and Biomedical Sciences, Colorado State University, Fort Collins, CO, United States of America; 2 Department of Pharmacology, University of Colorado School of Medicine, Aurora, CO, United States of America; 3 Department of Veterinary Population Medicine, College of Veterinary Medicine, University of Minnesota, St. Paul, MN, United States of America; 4 Center for Immune and Regenerative Medicine, Department of Clinical Sciences, Colorado State University, Fort Collins, CO, United States of America; Istituto di Ricerche Farmacologiche Mario Negri IRCCS, ITALY

## Abstract

Prion diseases are members of neurodegenerative protein misfolding diseases (NPMDs) that include Alzheimer’s, Parkinson’s and Huntington diseases, amyotrophic lateral sclerosis, tauopathies, traumatic brain injuries, and chronic traumatic encephalopathies. No known therapeutics extend survival or improve quality of life of humans afflicted with prion disease. We and others developed a new approach to NPMD therapy based on reducing the amount of the normal, host-encoded protein available as substrate for misfolding into pathologic forms, using RNA interference, a catabolic pathway that decreases levels of mRNA encoding a particular protein. We developed a therapeutic delivery system consisting of small interfering RNA (siRNA) complexed to liposomes and addressed to the central nervous system using a targeting peptide derived from rabies virus glycoprotein. These liposome-siRNA-peptide complexes (LSPCs) cross the blood-brain barrier and deliver PrP siRNA to neuronal cells to decrease expression of the normal cellular prion protein, PrP^C^, which acts as a substrate for prion replication. Here we show that LSPCs can extend survival and improve behavior of prion-infected mice that remain immunotolerant to treatment. LSPC treatment may be a viable therapy for prion and other NPMDs that can improve the quality of life of patients at terminal disease stages.

## Introduction

Prion diseases are neurodegenerative, protein misfolding diseases that have an impact on a broad range of species from sheep (scrapie), cervids (chronic wasting disease (CWD)) and cattle (bovine spongiform encephalopathy (BSE)) to humans (Creutzfeldt-Jakob disease (CJD), among others). Plaque deposits, neuronal vacuolation, glial activation, neuronal cell death and long incubation times characterize prion diseases [1–3]. Unlike other infectious agents, prions encode instructions for additional infectious particles within the structure of the misfolded form of a normal host protein, the cellular prion protein (PrP^C^), rather than nucleic acid sequence [4]. Consumption of infected meat or exposure to infected bodily fluids or tissues likely transmits infectious prion diseases, including scrapie, BSE, variant CJD in humans, and CWD [5,6]. However, spontaneous prion generation also results from several PrP^C^ polymorphisms that increase the likelihood of PrP^C^ misfolding. Most prion diseases can result from, or be influenced by *prnp* polymorphisms, including scrapie, BSE, CWD, genetic CJD, and fatal familial insomnia in humans [7,8].

PrP^C^, in its immature form, is a 250-amino acid protein that all mammals investigated express [9,10] throughout the body, with the highest levels of mRNA and protein detected in neurons of the central nervous system (CNS) [11,12]. PrP^C^ matures in the endoplasmic reticulum and the Golgi apparatus, where it is processed into a 208-amino acid protein with a glycosylphosphatidylinositol (GPI) anchor and two N-linked glycans [10,13]. Approximately half of the N-terminus of PrP^C^ adopts no formal structure, while the C-terminus folds into three α-helices and two short β-sheets. Its GPI anchor sequesters PrP^C^ within cholesterol and sphingolipid-rich rafts in the plasma membrane [14]. When PrP^C^ misfolds, it becomes known as PrP^Sc^ or PrP^Res^, which correlates with prion infectivity [4,15,16].

Research over the last 30 years revealed many proposed PrP^C^ functions. PrP^C^ can be neuroprotective through antioxidant [17–19] and anti-apoptotic functions [20,21]. The ability of PrP^C^ to regulate Ca^2+^ homeostasis [22–24], leading to activation of the MAPK/ERK, PKA, and STAT1 cell signaling pathways to modulate responses to oxidative and apoptotic damage [25,26] supports this neuroprotective function theory. PrP^C^ also binds to Cu^2+^ via tandem octapeptide repeats in the unstructured N-terminus, which is thought to mediate oxidative stress damage [27–29]. Other proposed PrP^C^ functions include hematopoietic stem cell renewal [30], axonal myelination sensing [31], immune activation [32,33] and regulation of circadian rhythms [34]. However, PrP-null mice develop, breed and behave normally [35], suggesting a redundant or inducible function for PrP^C^.

No known therapeutics improve quality of life or extend survival of humans afflicted with prion disease. Most early therapeutic compounds targeted conversion of PrP^C^ into PrP^Res^, including polyanionic compounds HPA-23 [36,37], dextran sulfate [36,38,39], pentosan polysulphate [39–41], and congo red [42–44]; and polycationic compounds, including branched polyamines [45,46], lipopolyamines [47] and dendrimers [48]. Antiviral [49–51], antibacterial [51–53], antimalarial [54–56] and an anti-cancer drug [57] have also shown therapeutic promise *in vitro*. Some of these compounds prolong survival in laboratory animals experimentally infected with prions, but showed little or no efficacy in human trials [58]. In recent years, anti-PrP antibodies administered as either active or passive immunization have also shown therapeutic promise [59–62], depending on the PrP epitope these antibodies recognize [63].

However, significant challenges remain before large-scale clinical trials are considered for these drugs. Most do not cross the blood-brain barrier (BBB) or target neuronal cells. Many polyionic compounds are toxic and cannot be given in large doses or over an extended time period. Some drugs only have anti-prion activity for specific prion strains. Most importantly, these compounds only have shown efficacy before or directly after prion inoculation, and few have shown any promise when given at late or clinical stages, when prion disease are typically diagnosed and invariably fatal.

We and others investigated a new approach to prion disease therapy based on the observation that 21% of mice heterozygous for the prion protein gene (*prnp*) and expressing approximately half the amount of PrP^C^ survived terminal prion disease, and the remaining mice lived 2.5 times longer than *prnp* homozygous mice [64–66]. We reasoned that therapeutics that reduce the PrP^C^ substrate required for prion replication by 50% should significantly prolong survival of prion-infected mice.

RNA interference (RNAi) is a catabolic pathway that utilizes RNA molecules to decrease levels of mRNA encoding a particular protein [67–70]. These RNA molecules, short hairpin RNA (shRNA) or small interfering RNA (siRNA), activate the RNA-induced silencing complex that cleaves mRNA and enables endo- and exonucleases to degrade the targeted mRNA resulting in a decrease in translated protein levels. We and others have shown that both shRNA and siRNA treatment targeted towards PrP^C^ can reduce the level of PrP^Res^ in cultured and primary cells by decreasing the amount of PrP^C^ available for conversion [71–73]. A single stereotactic injection of PrP shRNA into the hippocampus of prion-infected mice resulted in prolonged survival and reversal of prion neuropathology [72]. However, stereotactic injections are highly invasive and can compromise the BBB. Lentiviral delivery of shRNA also irreversibly silences *prnp* expression, affects a relatively localized brain region and still presents significant safety concerns. We have previously reported using liposome-siRNA-peptide complexes (LSPCs) addressed to nicotinic acetylcholine receptor (nAchR)- expressing cells using a short, modified peptide from rabies virus glycoprotein (RVG-9r) to deliver PrP siRNA to neuronal cells *in vitro* and *in vivo* and cure neuroblastoma cells chronically infected with prions [73]. We now focus on using LSPCs *in vivo* to treat prion-infected mice.

Here, we demonstrate that PrP siRNA LSPCs cross the BBB and decrease the amount of neuronal PrP^C^ 40–50% *in vivo* after a single intravascular injection in two different mouse models. We then treated prion-infected mice every two or four weeks starting midway through prion disease course with PrP siRNA LSPCs or control LSPCs and monitored mice for clinical and behavioral signs of prion disease. We found that repeated LSPC treatment significantly prolonged survival of 6/19 mice (responders) infected with prions, and significantly improved the behavior of all prion-infected mice at late stages of disease. Treated mice that did not survive significantly longer (non-responders) developed high anti-RVG-9r IgG titers, indicative of a significant immune response to the repetitive treatment protocol, while 5/6 responder mice did not seroconvert. Intranasal LSPC delivery doubled the response rate and decreased seroconversion rate by 50%. Limiting LSPC treatment to three transvascular injections prevented seroconversion, prolonged survival of PrP^C^ overexpressing mice up to 22%, and improved behavior of prion-infected mice compared to control-treated infected mice in this accelerated prion disease model. These results indicate that LSPC delivery of PrP siRNA significantly decreases PrP^C^ expression and subsequent prion replication in the brain that prolongs normal behavior and life span of prion-infected mice. *In* toto, this report promotes LSPCs, delivering siRNA targeting expression of normal host proteins that act as substrates for misfolding, as a viable candidate to treat prion diseases and other NPMDs.

## Results

### LSPCs deliver PrP^C^ siRNA to the brain

**Fig 1:**
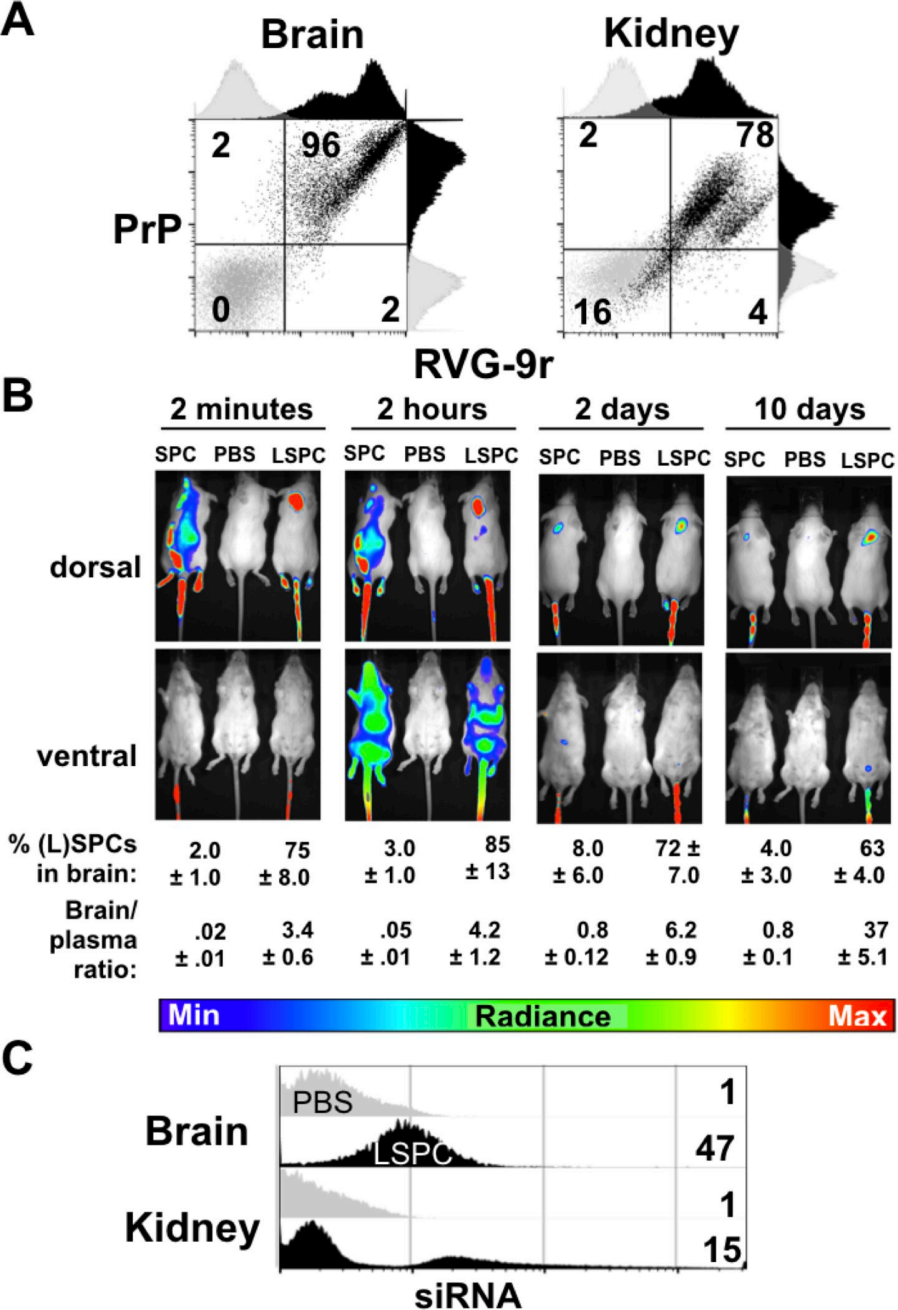
LSPCs target the majority of neuronal cells and deliver PrP^C^ siRNA to the brain when injected intravenously. A) Flow cytometry of neuronal cells labeled with BAR-224, an anti-PrP^C^ antibody, and RVG-9r reveals that 98% of cells in the brain display PrP^C^ and nAchRs on their surfaces. Ninety-six percent of those cells express both PrP^C^ and nAchRs. A smaller proportion of kidney cells (78%) express both PrP^C^ and nAchRs. B) *In vivo* live imaging revealed that RVG-9r increased LSPC delivery to the brain two minutes to ten days after intravascular injection compared to siRNA and peptide complexes without liposomes (SPCs). We controlled for background fluorescence using PBS-injected mice. C) Flow cytometry analysis 24 hours after fluorescent LSPC injection revealed siRNA delivery to 47% of brain cells and 15% of kidney cells (black histograms) compared to PBS-injected controls (gray histograms). Data are representative of at least three independent experiments.

To assess the number of targetable cells within the brain that express PrP^C^ and nAchRs, we incubated primary neuronal cells with RVG-9r and the anti-PrP^C^ antibody BAR-224 and performed flow cytometry. The majority (96.4%) of neuronal cells stained double-positive for BAR-224 and RVG-9r, indicating that most brain cells express both PrP^C^ and nAchR (Fig 1A) and are targets for PrP LSPCs addressed with RVG-9r. We previously reported RVG-9r binding to cultured kidney cells [74]. To assess possible off-target effects of LSPCs *in vivo*, we also incubated primary kidney cells with BAR-224 and RVG-9r. Flow cytometry revealed fewer kidney cells (80% versus 98%) expressing 10-fold less PrP^C^ and two-fold less nAchRs, indicating a diminished potential for LSPCs targeting the kidney compared to the brain.

We next combined *in vivo* whole animal imaging with flow cytometry to assess pharmacokinetics of LSPC delivery to the brain after intravascular injection. To observe the biodistribution of LSPCs, naïve FVB mice were injected intravenously with LSPCs containing Alexa 488-labeled PrP^C^ siRNA and RVG-9r labeled with Dylight 650. *In vivo* live imaging showed that mice injected intravenously with siRNA-peptide only complexes (SPCs) had a wide biodistribution and rapidly decreasing fluorescence within the body with little PrP^C^ siRNA signal in the brain, whereas mice injected intravenously with liposomes containing siRNA and the RVG-9r peptide (LSPCs) showed increased signal of PrP siRNA within the brain two minutes to ten days after injection (Fig 1B). Eighty-five percent of LSPCs were detected in the brain by two hours post injection, and 63% persisted in the brain ten days later. Flow cytometry of cellular targets in these mice revealed 47% of brain cells and 15% of kidney cells contained PrP siRNA (Fig 1C).

### Intravenous LSPC administration decreases neuronal PrP^C^ protein and mRNA levels

**Fig 2:**
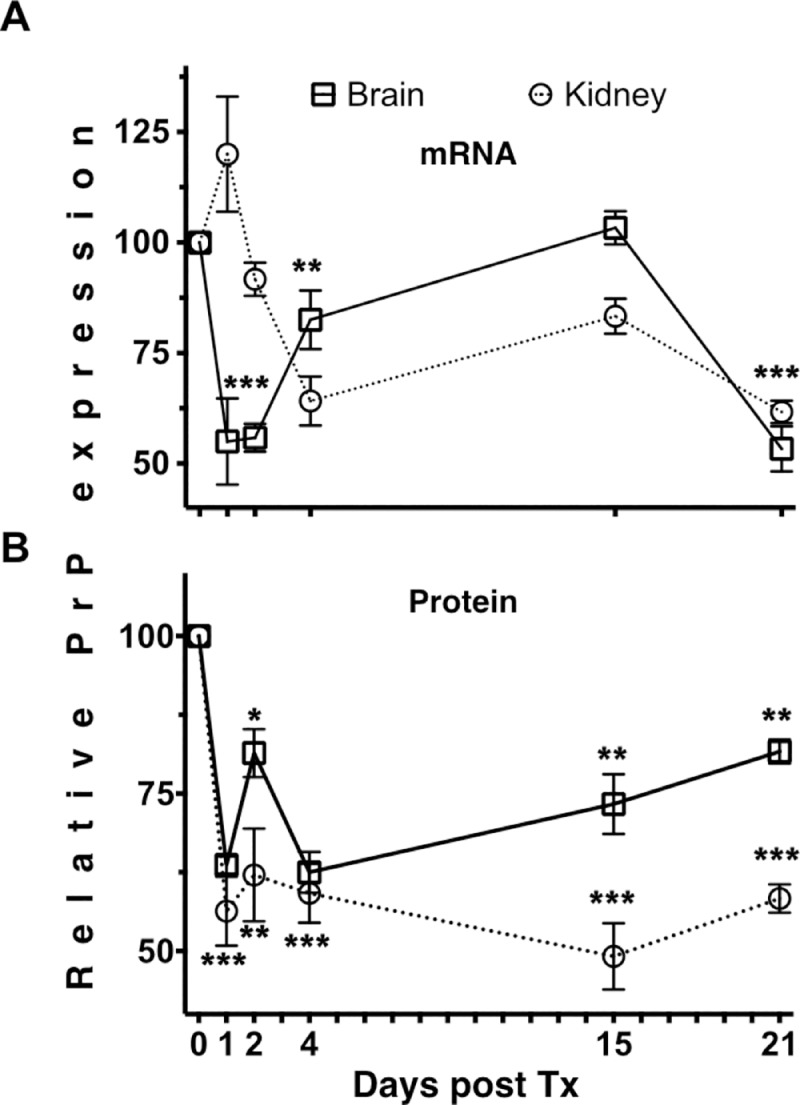
Intravenous treatment of naïve wild-type mice with PrP LSPCs decreased PrP^C^ mRNA and cell surface PrP^C^ protein at various time points. FVB (n = 7), CD-1 (n = 6) and C57Bl/6 mice (n = 5) were injected with LSPCs intravascularly. We assessed PrP^C^ mRNA and cell surface PrP^C^ expression via ddPCR and flow cytometry, respectively, in brain and kidney cells in three mice at each of the indicated time points after treatment. LSPCs reduced PrP (A) mRNA and (B) protein expression 20–50% in brains and kidneys of wild -type mice for up to 21 days after a single injection. Error bars indicate 95% confidence interval. * p<0.05, ** p<0.01, *** p<0.001, repeated measures ANOVA.

To assess pharmacodynamics of PrP knockdown via LSPCs, wild type mice were injected intravenously with LSPCs and monitored for mRNA expression and PrP^C^ protein levels at serial time points after treatment using digital droplet PCR (ddPCR) and flow cytometry, respectively. FVB, CD-1 and C57Bl/6 mice expressed decreased levels of neuronal PrP^C^ mRNA (Fig 2A) and protein (Fig 2B) at multiple time points in both brains and kidneys of LSPC-treated mice. Additional control experiments revealed that mice injected with LSPCs carrying irrelevant siRNA or addressed with a control peptide (RVM) that does not target nAchRs, expressed normal amounts of PrP^C^ mRNA and protein in both brains and kidneys at 4 days post treatment (S1 Fig). Mice injected with LSPCs carrying scrambled PrP siRNA expressed more PrP mRNA, but not protein, in brains; and more PrP mRNA and slightly less PrP protein in kidneys at 4 days post-treatment.

### Repetitive LSPC treatment prolongs survival in a subset of prion-infected mice

**Fig 3:**
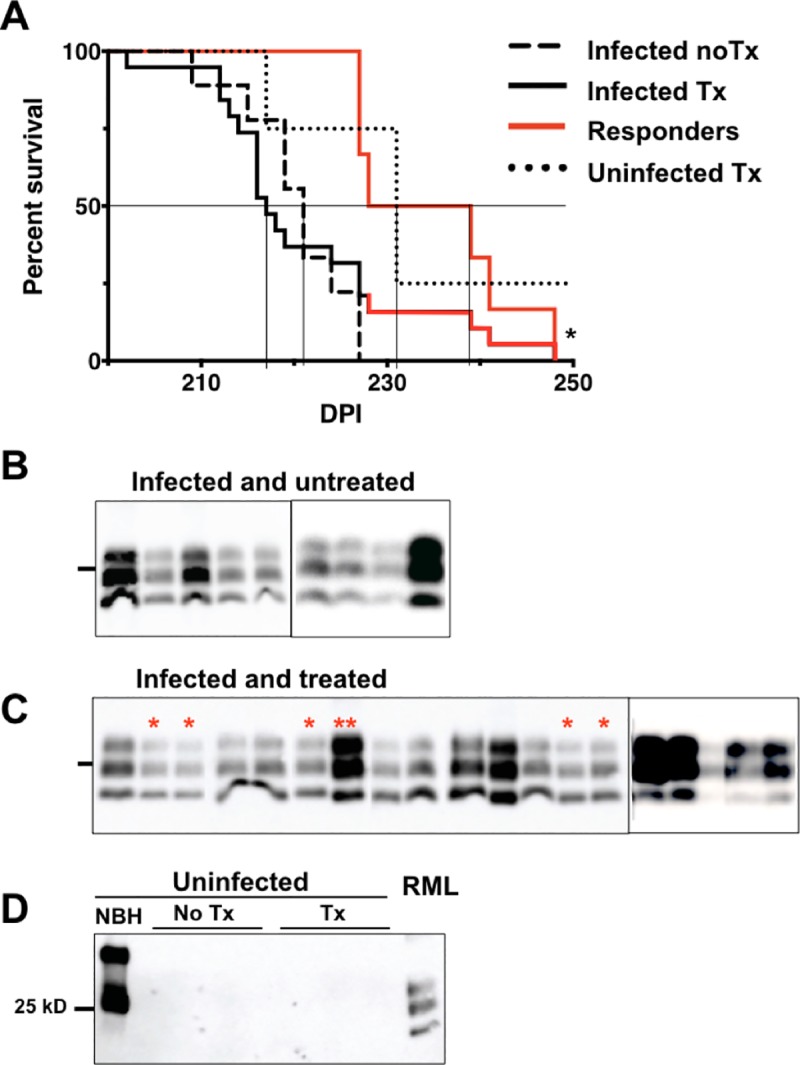
Repeated LSPC treatment prolongs survival of and decreases PrP^Res^ accumulation in a subset of prion-infected mice. Wild type mice were injected intraperitoneally with RML-5 prions and treated intravenously, intranasally, or both with 1578 PrP^C^ siRNA LSPCs or 1672 PrP^C^ siRNA LSPCs. We observed no significant differences in survival times among different routes or siRNA used, so we present compiled data here. See S3 Fig for survival curves for each delivery route. A) We observed no significant difference between control infected untreated mice (dashed black line, n = 9) and prion-infected, LSPCs-treated mice (solid black line, n = 19). However, a subset of treated mice positively responded to LSPC treatment (both solid red lines, n = 6, one shown extending from solid black line of all infected treated mice, the other as its own group), surviving significantly longer than non-responders and untreated mice (*p<0.05, survival analysis). Three of five uninfected treated mice (dotted black line) also died unexpectedly. All infected mice harbored PrP^Res^ (B and C). All samples were treated with 50 μg/mL PK. Red asterisks denote samples from responder mice, 5 of 6 of which appeared to contain relatively less PrP^Res^. Double asterisks denote responder #4. We detected no PrP^Res^ in uninfected (un)treated mice (D) All samples except lane 1 were treated with 50 μg/mL PK. Black lines to the left of each blot indicates the 25 kD molecular weight marker. NBH, normal brain homogenate. RML, Rocky Mountain Lab prion strain. Samples in all lanes were digested with 50 μg/mL Proteinase K, except NBH.

Based on these data, we treated prion-infected mice every two weeks with LSPCs and monitored them for behavioral, cognitive and clinical signs of prion pathogenesis. Because extraneural prion exposure more closely mimics a natural prion infection, we inoculated WT mice intraperitoneally with RML-5 prions, then began treating mice approximately midway through prion disease course (see S1 Table), when onset of early behavioral and cognitive changes occur. We delivered LSPCs intravenously (IV) to most of the mice in this study. However, a subset of mice received LSPCs intranasally (IN, n = 5) to assess efficacy of a less invasive route previously shown to be an effective method to deliver drugs to the brain [75,76]. An additional cohort received LSPCs via both routes (n = 5). We used LSPCs to deliver two PrP^C^ siRNAs, 1578 or 1672, that both targeted the 3’ untranslated region of the *prnp* locus [74]. Mice began exhibiting clinical signs of terminal prion disease after the seventh LSPC injection and were subsequently euthanized beginning 203 days post infection (DPI), while a minority of mice lived to receive nine (n = 6) LSPC treatments (> 217 DPI). We observed no difference in survival proportion or time between infected, treated (19/19 died with a mean survival time of 221, 95% confidence interval (CI) ± 5 DPI) and untreated mice (9/9, 220 ± 4 DPI, Fig 3A). However, 6 of 19 treated mice responded significantly better to treatment, surviving terminal prion disease significantly longer (235 ± 9 DPI) than non-responders (215 ± 3 DPI) and untreated mice. Of the 6 responder mice, 3 were treated IV (33% response rate) and lived to a mean survival time of 232 ± 5 DPI. The other 3 responders were treated IN (60% response rate) and survived a mean 238 ± 7 DPI (p < 0.05, S2 Fig). Mice treated by both routes simultaneously did not survive significantly longer than untreated or non-responder mice (212 ± 8 DPI).

**Fig 4:**
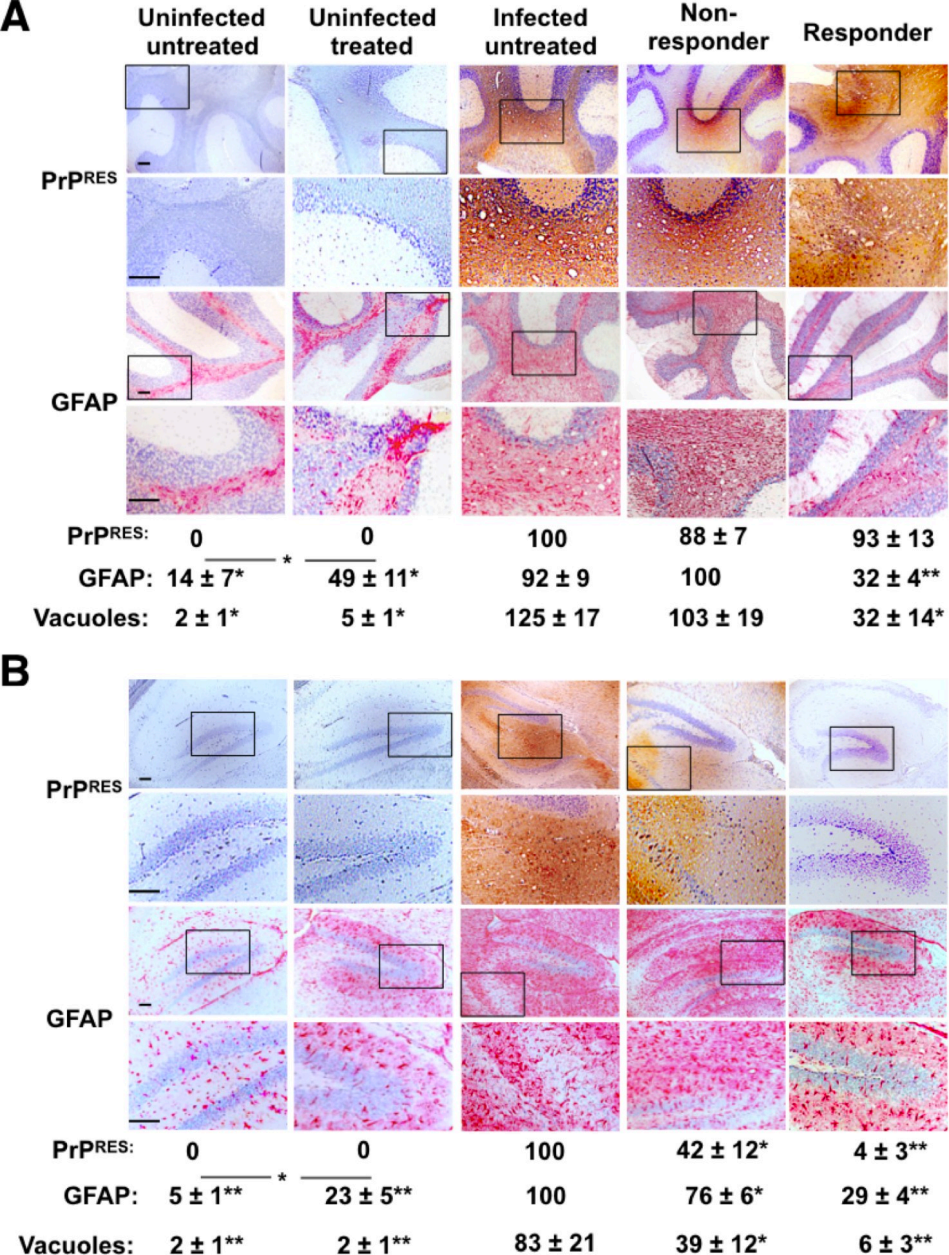
LSPC treatment decreases neuropathology in prion-infected mice. Immunohistochemistry (IHC) of brain sections through the (A) cerebellum and (B) hippocampus in infected and uninfected mice reveal that (A) while we observed no difference in cerebellar PrP^Res^ accumulation among infected untreated, non-responder or responder mice, both GFAP stain intensity, a measure of astrogliosis, and vacuolation, a hallmark of prion-mediated neurodegeneration, were significantly decreased in LSPC responder mice. (B) In the hippocampus, we observed decreased PrP^Res^, GFAP and vacuolation in responder and non-responder mice compared to infected untreated mice. We detected no PrP^Res^ or vacuolation but significant GFAP expression in both cerebellum and hippocampus of uninfected treated mice compared to uninfected untreated mice. Boxed areas indicate magnified areas in the panel directly below. Scale bars, 100 μm. Quantitation of PrP^Res^ and GFAP is expressed as relative pixel intensity ± 95% CI per mm^2^. Vacuolation scores indicate number of vacuoles ± 95% CI per mm^2^. IHC images are representative of at least three mice per group. We collected data from at least three non-consecutive slides from at least two animals from each group. *p < 0.05, **p < 0.01, compared to scores from infected untreated mice, except where indicated otherwise.

Proteinase K digestion and western blot analysis of brains from prion-infected mice showed PrP^Res^ deposition at terminal disease, with brains from 5 of 6 responder animals appearing to harbor relatively less PrP^Res^ than most non-responders or infected untreated controls, although densitometry revealed these differences to be not quite statistically significant (p = 0.057 and 0.072, respectively, Fig 3B and 3C). Consistently, responder mice exhibited significantly reduced vacuolation and astrogliosis in the cerebellum than non-responders and infected untreated control mice, although PrP^Res^ accumulation was equivalent among the groups (Fig 4A). We observed more dramatic differences in the hippocampus, where we observed significantly less PrP^Res^, GFAP and vacuoles in both responders and non-responders compared to infected, untreated mice (Fig 4B). We observed variable and inconsistent neuropathology in other brain regions with or without of treatment.

### Repetitive LSPC treatment prolongs normal behavior in prion-infected mice

**Fig 5:**
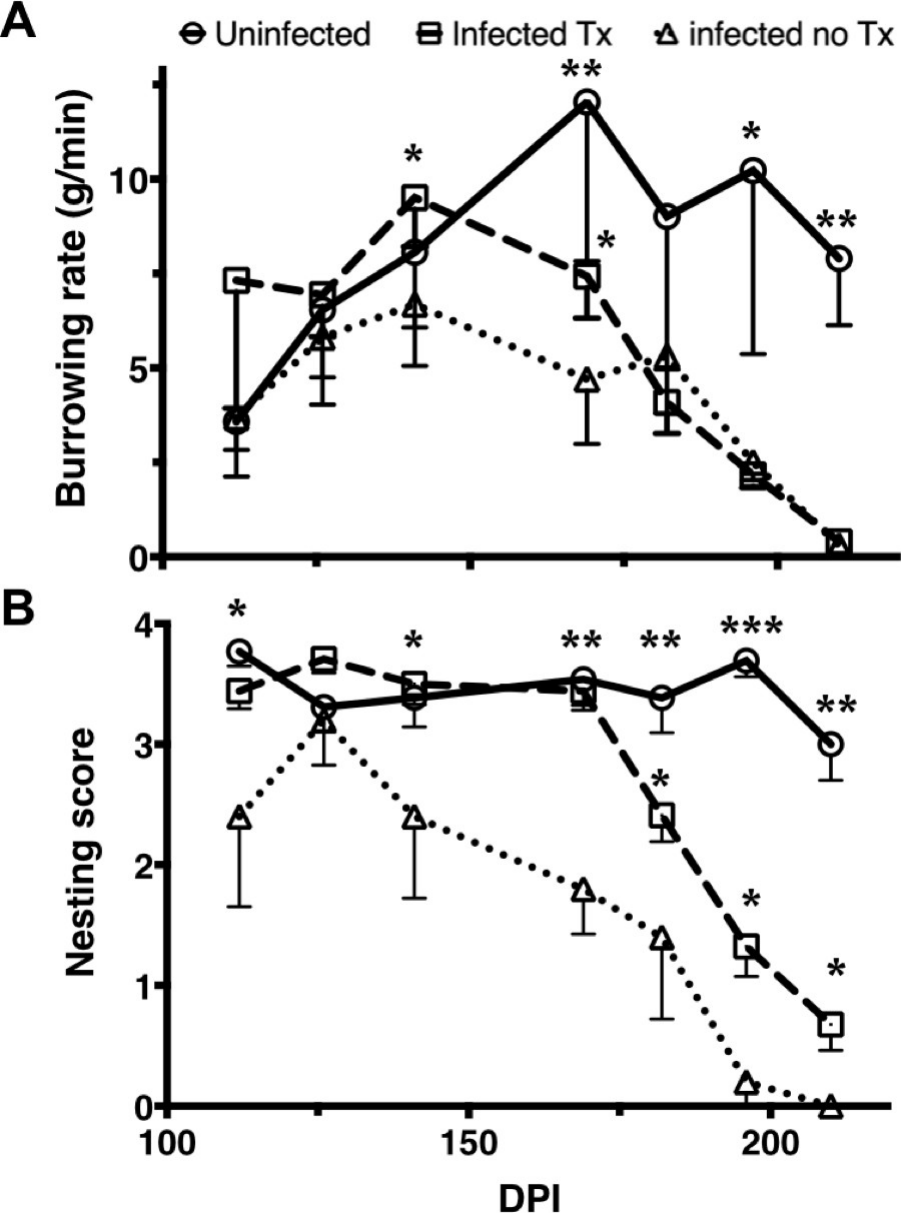
Prion-infected LSPC-treated mice have improved behavior scores compared to infected, untreated mice. We instituted longitudinal burrowing and nesting tests to assess behavioral changes in prion-infected mice starting four weeks before the first LSPC treatment. A) PrP siRNA LSPC treatment prolonged normal burrowing behavior in infected mice. B) We observed even more significant improvement in nesting behavior in infected mice treated with PrP siRNA LSPCs. Error bars indicate 95% confidence intervals. *p<0.05, ** p<0.01, *** p<0.001, two-way ANOVA.

We subjected all mice to behavioral testing to determine if LSPC treatment abrogates early behavioral deficits observed in prion-infected mice. We started burrowing and nesting tests four weeks before LSPC treatment and continued every two weeks thereafter until terminal disease or end of the study. While we observed differences in survival and neuropathology among treated mice (responders versus non-responders), we observed no significant differences in behavior between responders and non-responders. We also observed no differences in LSPC-treated or untreated, male or female mice. We therefore compared all treated mice to the infected untreated control group. PrP siRNA LSPC treatment prolonged normal burrowing rates (Fig 5A) and nesting scores (Fig 5B) up to 169 DPI in infected mice compared to untreated infected mice. LSPC treatment improved nesting in prion-infected mice up to terminal disease compared to untreated infected mice, although not to normal levels observed for uninfected treated mice (Fig 5B). These data indicate that PrP siRNA LSPC treatment can prolong normal behavior, and significantly improve behavior of prion-infected mice up to terminal disease.

### Anti-RVG-9r IgG detected in the serum of mice treated with LSPCs

**Fig 6:**
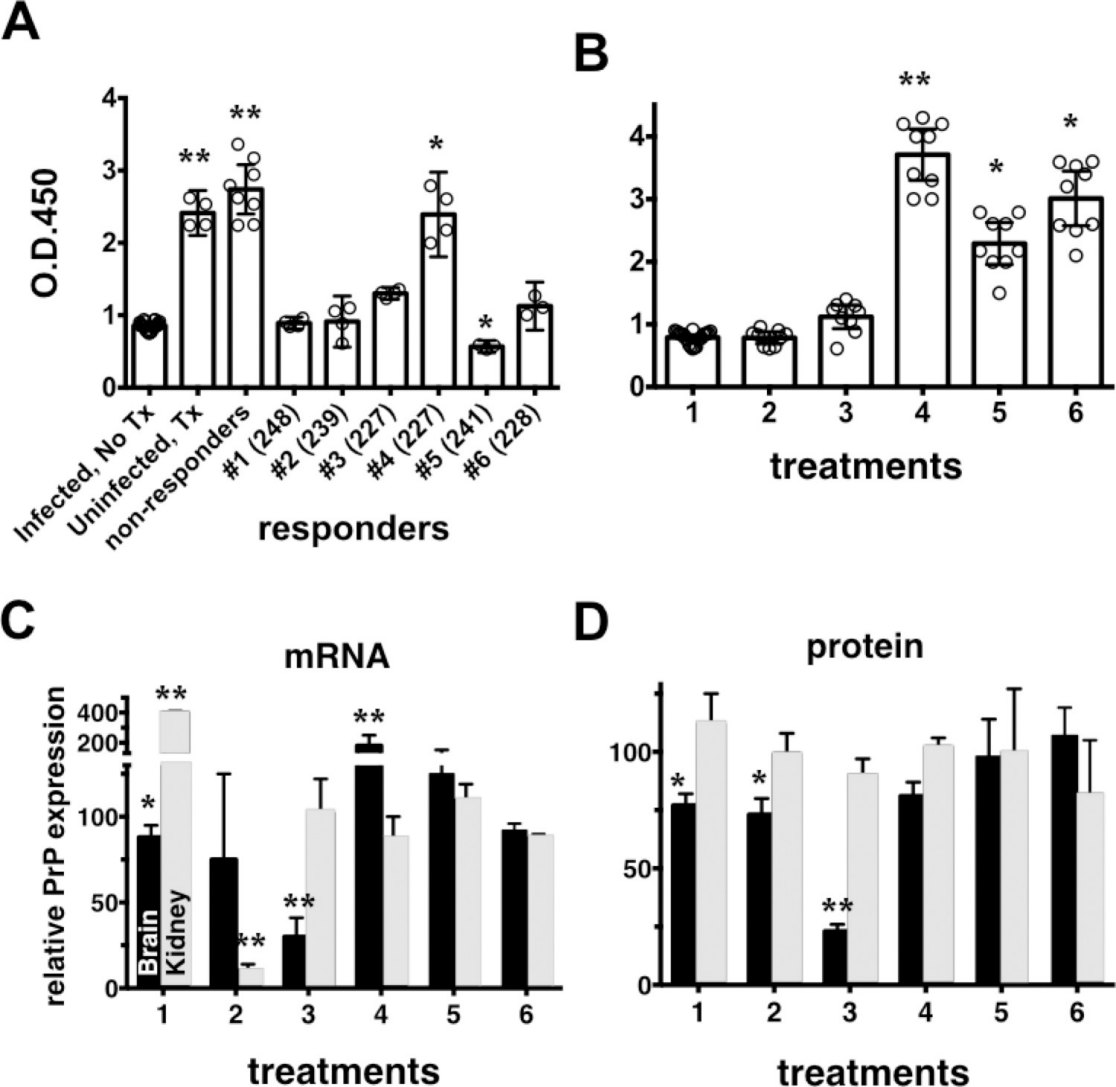
Significant anti-RVG-9r IgG titers detected in mice repeatedly treated with LSPCs, but not in responder mice, coincide with loss of PrP^C^ knockdown. A) Total IgG levels, measured using an ELISA assay, against RVG-9r. We detected significant IgG titers against the RVG-9r peptide in uninfected treated and non-responder mice and one responder (#4) mouse. Error bars indicate 95% confidence intervals. (B) We detected significant anti-RVG-9r IgG titers in mice beginning after the fourth LSPC treatment (B) that coincide with de-repression of PrP mRNA (C) and protein (D) expression. * p < 0.05, ** p < 0.01, one-way and two-way ANOVA.

Unexpectedly, 3 of 4 uninfected, LSPC-treated control mice died suddenly one hour after the ninth LSPC treatment, indicating possible toxicity of repeated LSPC administration, and were immediately and humanely euthanized. Neuropathology, including some vacuolation and abundant astrogliosis, in the absence of PrP^Res^ deposition in the brains of these mice (Fig 4A and 4B), corroborate this hypothesis. We did not observe acute, unexpected deaths in any other infected, treated or untreated mice. Given that a majority of uninfected LSPC-treated mice died at relatively young ages (287–329 days old), we reasoned that pathogenesis resulting from immune responses against LSPCs may have masked therapeutic benefit of LSPCs against prion disease. Necropsy of these control mice revealed enlarged, darkened spleens and kidneys and severe blood coagulation, indicative of extensive immune complex formation likely contributing to death of these animals. To determine the extent of immune activation against LSPCs, we collected serum samples from mice at time of euthanasia and measured anti-RVG-9r IgG levels by indirect ELISA. We detected significant anti-RVG-9r IgG titers in all uninfected treated (n = 4) and infected, LSPC non-responsive mice (n = 13, Fig 6A). Five of 6 LSPC-responsive mice and all untreated, RVG-9r naïve mice produced no significant anti-RVG-9r IgG titers. (Fig 6A). Only one IV-treated responder mouse (#4), the same mouse with increased PrP^Res^ (Fig 3C, two red asterisks), produced significant anti-RVG-9r IgG titers. We initiated a second independent experiment, limiting treatment of mice to 4 LSPC injections 24 to 33 days apart to avoid immune response to treatment. We observed no significant difference in survival times between infected, untreated mice (223 ± 3 DPI, n = 13) and infected treated mice (219 ± 2, n = 18).

### More than three LSPC treatments increase anti-RVG-9r IgG levels and abrogates PrP^C^ knockdown

We next sought to determine the number of LSPC treatments that would result in the maximal decrease of PrP^C^ protein and mRNA and minimal anti-RVG-9r IgG titers. We infected WT mice intraperitoneally with RML-5 prions then treated with LSPCs every two weeks for a total of ten weeks. After every treatment, we euthanized groups of treated and untreated mice (n = 5 per group) and analyzed their sera for anti-RVG-9r IgG titers, and brains and kidneys for PrP mRNA and protein expression. We detected no significant RVG-specific IgG titers in any of the LSPC-treated mice until after the third LSPC treatment, after which IgG titers rose steadily (Fig 6B). We observed maximal decrease of PrP mRNA and protein up to three repetitive LSPC treatments every two weeks, which resulted in a 3-fold decrease of neuronal PrP^C^ levels (Fig 6C and 6D). After three treatments, PrP mRNA and protein levels steadily increased in the brain (Fig 6C and 6D), concomitant with detection of increasing RVG-9r-specific IgG titers (Fig 6B). Messenger RNA and protein levels in the kidney were more variable but followed a similar trend: initial decrease in PrP mRNA (after 2 treatments) and protein (after 3 treatments), followed by a steady rise to normal levels after subsequent treatments. These results strongly suggest that an immunological response to LSPCs *in vivo* abrogates their therapeutic effect.

### Limited PrP siRNA LSPC treatment extends survival time and normal behavior in an accelerated prion disease model

**Fig 7:**
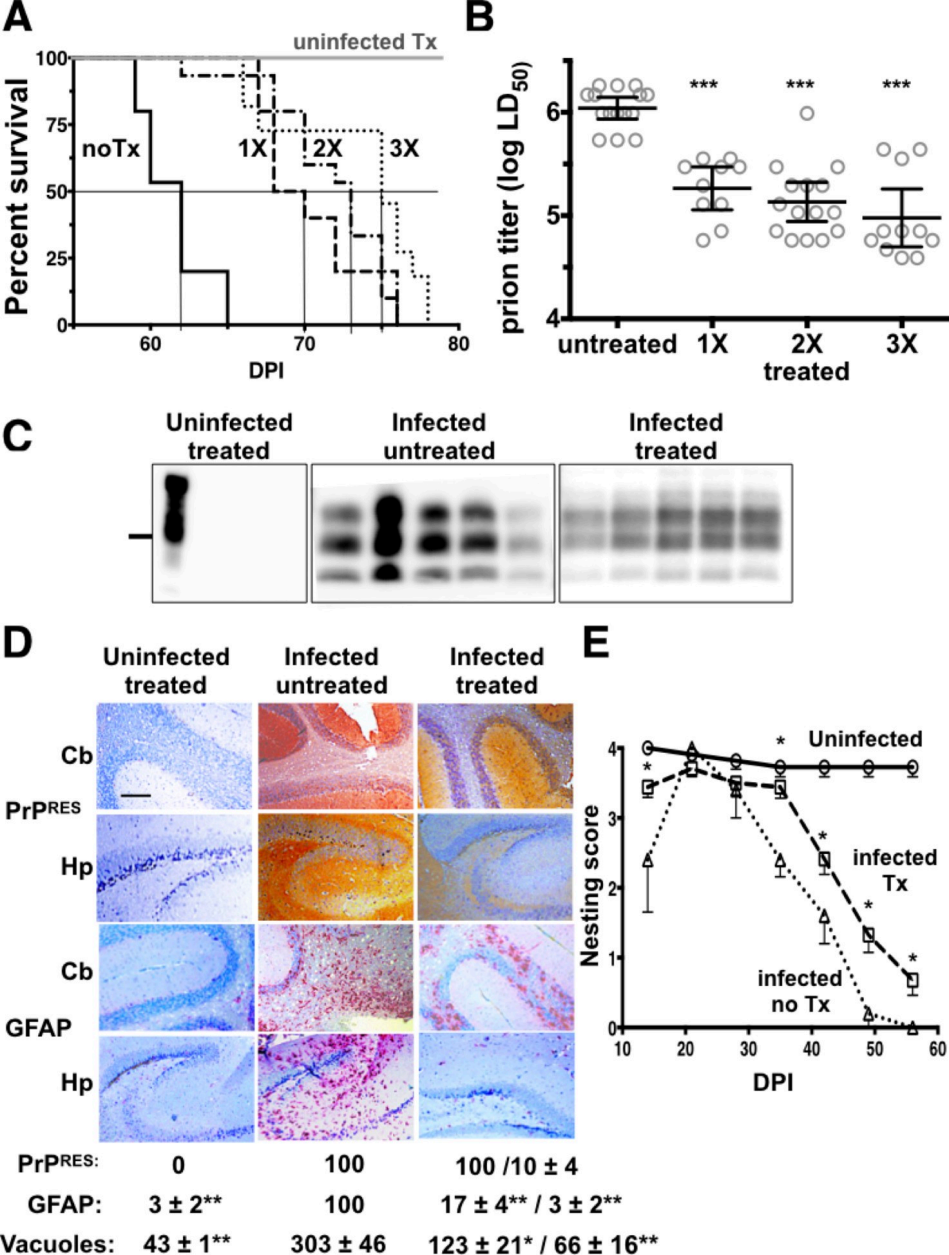
Limited LSPC treatment extends survival times and normal nesting behavior in an accelerated prion disease mouse model. (A). Survival of prion infected TgA20 mice was significantly extended with just one PrP siRNA LSPC treatment given 40 DPI (dashed line), and extended further with two (dashed dotted line, given 40 and 60 DPI) and three (dotted line, given 20, 40 and 60 DPI) treatments over infected untreated mice (solid black line). All uninfected treated mice appeared normal and survived over 100 days after three treatments (solid gray line). (B) LSPC treatment reduced prion replication up to 90%. All mice were infected with 10^6^ LD_50_ units of RML-5 prions. The graph shows equivalent prion titers based on time to terminal disease. (C) Representative immunoblot showing infected, LSPC treated mice appeared to harbor less PrP^Res^ in their brains compared to brain samples from infected untreated mice, although densitometry revealed no significant differences (p = 0.074), except in uninfected treated mice, which harbored no PrP^Res^. All samples except lane 1 were treated with 50 μg/mL PK. (D). IHC revealed no PrP^Res^ and little to no GFAP reactivity in uninfected mice treated three times with LSPCs (first column). While we observed no difference in cerebellar (Cb) PrP^Res^ accumulation in infected mice untreated (second column) or treated (third column) with LSPCs, we did observe significantly less PrP^Res^ in the hippocampus (Hp) of infected, treated mice compared to infected, untreated mice. We also observed significantly less GFAP and vacuoles overall in brains of infected and uninfected, treated mice compared to infected untreated mice. Scale bar, 100 μm. Quantitation of infected treated samples (third column) show values for Cb/Hp. All other values are combined Cb and Hp scores. We collected data from at least three non-consecutive sections from at least two animals from each group. (E) Combined nesting scores of all treatment groups revealed prolonged, significantly improved nesting behavior in treated mice (dashed line with squares) compared to infected untreated mice (dotted line and triangles). Uninfected treated mice sustained normal behavior for the duration of the study (solid gray line). *p < 0.05, **p < 0.01, ***p < 0.001 compared to values from infected untreated mice.

Given that immune responses to the RVG-9r targeting peptide after three exposures limits LSPC efficacy, we performed an additional therapeutic study using an accelerated prion disease mouse model. TgA20 mice express 4-7-fold more PrP^C^, and succumb to prion disease 4–5 times faster than wild-type mice [77]. TgA20 mice are also the gold standard for titering the RML-5 prion strain [65,78,79], so we can accurately assess prion replication with or without LSPC treatment. We leveraged accelerated disease kinetics and precise prion titration to determine whether LSPCs, in the absence of an immune response against them, could extend survival and normal behavior by reducing prion replication and subsequent neuropathology that we observed in a typical prion disease course. We infected TgA20 mice intracerebrally with 10^6^ LD_50_ units of RML-5 prions, then beginning at 20 DPI treated them 1–3 times with PrP siRNA LSPC treatments 20 days apart. We detected no anti-RVG-9r IgG titers in any treated mice using this regimen (S3 Fig). While all infected mice still succumbed to terminal prion disease, we observed a significant 15–22% increase in mean survival time in infected mice treated once (70 ± 95% CI ± 1 DPI, n = 10), twice (72 ± 2 DPI, n = 15) or thrice (74 ± 1 DPI, n = 11) with LSPCs compared to untreated infected mice (61 ± 1 DPI, n = 15, p < 0.01, Fig 7A). Delays to terminal disease in infected treated mice equate to approximately one log less prion infectivity, equivalent to approximately 90% reduction in prion replication (Fig 7B). We detected PrP^Res^ in brain homogenates from all infected mice but not uninfected treated controls (Fig 7C). Immunohistochemistry (IHC) revealed similar PrP^Res^ accumulation in the cerebellum of all prion-infected mice, but significantly reduced PrP^Res^ in the hippocampus of infected treated mice compared to untreated mice (Fig 7D). We also observed far less GFAP signal and vacuolation in the cerebellum and hippocampus of infected and uninfected treated mice compared to infected untreated mice. Normal nesting behavior was prolonged to midway through disease course in infected treated mice compared to infected untreated mice (Fig 7D). After this point, we observed impaired nesting behavior in prion-infected mice compare to uninfected controls, although LSPC-treated mice did exhibit improved nesting behavior compared to untreated mice.

## Discussion

The blood-brain barrier (BBB) is a physical barrier composed of endothelial cells, pericytes, and astrocytes that protects neuronal cell types from infection, serum proteins and toxins. Crossing the BBB is the rate-limiting step for delivery of therapeutics to the brain and remains the biggest challenge in producing effective therapeutics for neurodegenerative disorders. The BBB prevents access to the central nervous system (CNS) in a number of ways, including tight junctions between endothelial cells, efflux pumps and cell-surface proteases [80–82].

While the BBB is formidable, therapeutic drugs can still cross this membrane if designed appropriately. Many strategies have been employed to transport drugs across the BBB, but the ‘Trojan Horse’ method is perhaps the best-known approach. A targeting ligand bound to a drug or delivery system binds to a cell-surface receptor on endothelial cells, actively transporting the drug across the BBB. Care must be taken to avoid the endocytotic pathway of the endothelial cells in favor of the transcytotic pathway for transport [80,81]. Multiple ligands and their cognate receptors have been used with varying degrees of success to transport drugs and delivery systems across the BBB, including transferrin [83], insulin [84], low-density lipoprotein [85], low-density lipoprotein receptor ligands [86], leptin [87], and brain-derived neurotrophic factor [81].

Here, we used the neuro-targeting peptide RVG-9r to guide our therapeutic PrP^C^ siRNA across the BBB. RVG-9r is a small peptide from the rabies virus glycoprotein that binds to the α7 subunit of nAchRs. Kumar et al. showed that RVG-9r dramatically increased siRNA delivery to the CNS when complexed together. siRNA bound to RVG-9r decreased exogenous GFP by 40%, and anti-viral siRNA against Japanese encephalitis virus increased survival times in mice infected with the virus [88]. We protect both PrP siRNA and RVG-9r in our formulation by complexing them with liposomes to increase serum half-life and decrease nuclease attack by serum proteins. We have previously characterized the ability of our LSPCs to deliver PrP^C^ siRNA to neuronal cells *in vitro* [73]. LSPCs delivered PrP siRNA directly to mouse neuroblastoma cells without the need for lipofection reagents and decreased PrP^C^ levels by 50–75% in these cells. The liposomes protected the siRNA from serum degradation, while the RVG-9r peptide delivered PrP^C^ siRNA specifically to cells that expressed nAchRs and decreased PrP^C^ expression, both *in vitro* and *in vivo* [73]. In this report, we confirmed these preliminary *in vivo* results and optimized LSPC dosing regimen to treat prion-infected mice.

We evaluated LSPC pharmacodynamics in two inbred wild-type mouse strains, C57Bl/6 and FVB, and outbred CD-1 mice. Most of the PrP siRNA LSPCs reached the brain by two hours post-injection and remained there for at least 10 days. LSPCs decreased neuronal and renal PrP^C^ mRNA and protein in both mouse lines by up to 50%. We observed no overt biological effects of decreasing PrP^C^ in kidneys, which normally express 10-fold less PrP^C^ and 2-fold less nAChRs than brain cells. However, prudence dictates monitoring these off-target effects in future therapeutic trials.

We first performed a two-week treatment regimen of prion-infected mice given that neuronal PrP^C^ is decreased from four to fifteen days after a single LSPC injection. We also opted to start our LSPC therapeutic midway through prion disease course to determine whether the treatment could reverse the early neuronal changes seen in prion-infected mice. When we observed that repeated LSPC injections every two weeks were causing significant side effects, we performed another LSPC treatment study with intervals ranging from 22–33 days. Unfortunately, repeated LSPC treatments at extended intervals did not extend the survival times of prion-infected mice. However, we discovered a subset (6/19) of infected mice treated every two weeks responded positively to LSPC therapy, living approximately 7% longer than infected untreated control and 9% longer than infected, non-responder treated mice. Five of six responder mice appeared to accumulate less PrP^Res^ and GFAP, and contained fewer vacuoles in their brains compared to infected untreated and non-responder mice. We observed a more pronounced decrease in neuropathology in the hippocampus, involved in learning, memory and behavior; compared to the cerebellum, which functions in motor control and coordination. These neuropathologic observations correlate with behavioral assessments revealing prolonged, improved burrowing and nesting in all infected mice treated with LSPCs, including non-responders, over infected untreated controls. Improvement in nesting, but not burrowing behavior, was sustained until terminal disease. More severe destruction of the cerebellum may have impaired the ability of treated mice to coordinate movement required for burrowing, while nesting requires less motor coordination and strength. Improvements in these behavioral tests, which model human activities of daily living and social engagement [89–92], may be an important predictor of therapeutic success for LSPCs and other drugs combating neurodegenerative diseases. That LSPC treatment improved behavior may be important for at least sustaining quality of life for afflicted individuals, if not extending survival time.

Deaths of the uninfected control group were unexpected. Three of four mice in this group died or were euthanized one hour after LSPC treatment due to severe morbidity. Observations noted during necropsy and the sudden morbidity after treatment suggested that these mice died of an acute Type III immune complex response. Total IgG levels against RVG-9r were increased in most treated mice, indicating that RVG-9r is stimulating the immune system. Increased astrogliosis in these mice supports this contention. However, 5 of 6 responder mice expressed no significant anti-RVG-9r titers and reduced astrogliosis. Immune responses against the RVG-9r peptide were not observed previously [88], but did not entail repeated administrations utilized here. Indeed, we did not detect anti-RVG-9r titers until after the third exposure, consistent with potentiating an immune response. Moreover, PrP siRNA may act as a TLR agonist, especially when complexed with liposomes, creating a powerful adjuvant for antibody production [93,94].

Scientists developing LSPCs and similar therapeutics with immunogenic potential must consider strategies to avoid or circumvent these immune responses. Here we tested IN LSPC delivery as a more direct route to the central nervous system that avoids LSPC exposure to the spleen and most other peripheral lymph nodes en route. We observed double the response rate (60 versus 33%) and half the seroconversion rate (40 versus 78%) among IN versus IV treated mice, respectively. Simultaneous IV and IN treatment potentiated seroconversion to 100%, suggesting spatiotemporal cross-priming and further emphasizing the need to monitor and control unwanted immune responses to LSPCs. We are currently exploring transient immunosuppression during IN delivery to circumvent the immune response that seemed to dampen therapeutic benefit of LSPCs. We are also exploring liposome modifications, include adding PEGylated groups to make them less available to bind to serum proteins and generate immune complexes [95,96].

To investigate whether this immune-mediated suppression of LSPC can be circumvented, we conducted a third LSPC treatment study using an accelerated disease progression model wherein we can administer fewer LSPCs treatments. We intracerebrally inoculated TgA20 mice, who die from prion infection in 60 days, with prions and treated them once, twice, or thrice. As expected, three LSPC treatments did not induce anti-RVG-9r titers but did result in significantly prolonged survival (up to 22%) and improved behavior that correlated with decreased neuropathology in treated mice compared to untreated mice. These results are consistent with previous studies using lentiviral delivery directly to the brain via stereotactic injection [72] (up to 24%), while using a far less invasive and potentially safer delivery system, if modifications to avoid unwanted immune responses can be achieved. LSPC treatment also impaired prion replication in the brains of infected TgA20 mice by up to 90%. A peculiarity of prion disease is that accumulation of PrP^Res^, as assessed by PK digestion and western blotting, does not always correlate to infectious prion titers, as assessed by mouse bioassay [97–102]. This anomaly could explain why LSPC treated mice replicated less prions and lived longer despite detecting no significant difference in PrP^Res^. Additionally, PrP^Res^ distribution in infected mice is not uniform: we detected most PrP^Res^ in the cerebellum, and far less in the hippocampus.

In summary, these data support LSPCs as an efficient vehicle to deliver therapeutic drugs across the BBB to the CNS. Additionally, the siRNA knockdown of PrP^C^, the substrate for prion replication, can reduce prion replication and neuropathology, and extend survival times in RVG-9r immunotolerant mice. Importantly, LSPC therapy may also significantly prolong normal behavior, and ameliorate cognitive decline associated with prion diseases. LSPC therapy could be extended to treat other protein misfolding diseases, like Alzheimer’s, Parkinson’s and Huntington Diseases, and amyotrophic lateral sclerosis. Therapeutics that can improve quality of life and daily living activities of afflicted patients would represent tremendous progress in treating these increasingly prevalent diseases. However, immune responses to these therapeutics must be carefully considered and avoided to prevent exacerbating disease-mediated neuropathology with immune-mediated neuroinflammation.

## Materials and methods

### Mice

C57Bl/6, FVB and CD-1 mice were purchased from The Jackson Laboratory (Bar Harbor, ME). TgA20 mice overexpressing PrP^C^ were created as previously described [77]. Mice were euthanized using CO_2_. All mice were bred and maintained at Lab Animal Resources, accredited by the Association for Assessment and Accreditation of Lab Animal Care International, in accordance with protocols approved by the Institutional Animal Care and Use Committee at Colorado State University.

### Generation of liposomes

1,2-dioleoyl-3-trimethylammonium-propane (DOTAP) LSPCs consist of a 1:1 DOTAP:cholesterol (Avanti Lipids 890890 and 700000) ratio in a 1:1 chloroform:methanol solution. The solvents were evaporated using N_2_ gas and the resultant dry lipid film was placed under vacuum for a total of 8 hours to remove any excess solvent. A stock solution of liposomes was made at an 8 mM (40 μmole total) concentration by resuspending the lipid film in 5 mL of 10% sucrose heated at 55°C. All components (lipid film and sucrose) were kept at this temperature during rehydration. One milliliter of heated sucrose was added to the lipid cake every 10 minutes and the lipid film swirled every 3 minutes to promote lipid mixing. Resulting liposomes were stored at 4°C.

### Generating LSPCs and treating mice

PrP^C^ 1578 siRNA sequence: GAAGTAGGCTCCATTCCAAA (Qiagen)

PrP^C^ 1672 siRNA sequence: ACATAAACTGCGATAGCTTC (Qiagen).

RVG-9r peptide: YTIWMPENPRPGTPCDIFTNSRGKRASNGGGGrrrrrrrrr (ChemPeptide)

DOTAP liposomes were diluted 1:100 in 1X PBS and sonicated 4X with 2–3 minute rests. Four nmol of diluted/sonicated liposomes was mixed with 4 nmole of 1672 siRNA. The siRNA/liposome solution was incubated for 10 minutes at 4°C. Then, 40 nmole of RVG-9r peptide was added to the solution and allowed to incubate for 10 minutes on ice. Mice were placed under a heat lamp for 5 minutes and anesthetized with 1.5–2% isofluorane (VetOne). Mouse tails were disinfected using 70% EtOH. LSPCs were injected into the tail veins of mice using a 29-gauge insulin syringe (BD Biosciences) or instilled into the nares of the nose using a pipette tip.

### Flow cytometry

One-half hemisphere of brain and one kidney was pressed through a 40 μm cell strainer (Falcon, VWR) using 5 mL of FACS buffer (1X PBS, 1% fetal bovine serum, 10 mM EDTA). Cells were washed 3X with PBS, centrifuged at 250x*g* and resuspended in FACS buffer (10% FBS, 1mM EDTA in PBS). Fc receptors were blocked using a 1:100 dilution of a 0.5 mg/mL rat anti-mouse CD16/CD32 Fc block (BD Biosciences) in FACS buffer with 7% goat serum for 20 minutes on ice and washed once as above. The cells were stained with a 1:100 dilution of 20 μg/mL solution of the PrP^C^ antibody BAR-224 conjugated to Dylight 650 (per manufacturer’s instructions, ThermoFisher) in FACS buffer for 40 minutes at room temperature. Red blood cells were lysed using RBC lysis buffer (1X PBS, 155 mM NH_4_Cl, 12 mM NaHCO_3_, 0.1 mM EDTA) for 3 minutes and then centrifuged at 250x*g* for 3 minutes. Cells were washed 2X with FACS buffer then stained with propidium iodide (Fisher Scientific) 10–15 minutes before analyzing a 1:2 dilution of the cells on a DakoCytomation Cyan ADP flow cytometer. Results were evaluated using FlowJo version 10.

### *In vivo* live imaging

LSPCs were assembled as described above, substituting PrP siRNA labeled with Alexa Fluor 488 (Qiagen) and RVG-9r labeled with Dylight 650 (per manufacturer’s instructions, ThermoFisher). Mice were anesthetized with 2% isofluorane, injected intravascularly with fluorescent LSPCs through the tail vein, and imaged using an IVIS Spectrum *in vivo* live imaging system. Autoexposure settings were used. siRNA signal was viewed with a 500/540 nm filter and RVG-9r signal was viewed with a 640/680 nm filter.

### RNA isolation, cDNA synthesis and digital droplet PCR (ddPCR)

RNA was extracted from brain and kidney cell suspensions using a RNeasy minikit (Qiagen). DNase digestion was performed off-column using RQ1 RNase-free DNase (Promega) per manufacturer’s instructions. We precipitated RNA from the DNase by adding 0.2 M Na Acetate and 2.5X volume of ice-cold 100% EtOH, then centrifuging at 16,060x*g* for 45 minutes. The RNA pellet was washed with 100% EtOH and centrifuged again at the same conditions. The RNA pellet was allowed to dry for 1 hour and was resuspended in molecular grade H_2_O. RNA concentration was assessed via spectrophotometry (Denovix) at 260 nm. Approximately 150 ng of RNA was used to generate cDNA using the High Capacity cDNA Reverse Transcription kit from ThermoFisher. A final concentration of 0.035 ng of cDNA and 1.25 μM of the following PrP primers was used in the ddPCR reactions: forward primer 5’CCTTGGTGGCTACATGCTGG-3’ and reverse primer 5’-GGCCTGTAGTACACTTGG-3’. Actin was used as an internal control: forward primer 5′-GACCTGACAGACTACCTCAT-3′ and reverse primer 5′-AGACAGCACTGTGTTGGCAT-3′. Supermix (BioRad) was added to the cDNA/primer solution to generate a final reaction volume of 20 μL. Droplet generator oil (BioRad) was added to the reaction mix and droplets were generated using a QX-100 droplet generator. Droplets were transferred to a 96-well plate and sealed with pierceable sealing foil sheets (BioRad). PCR amplification was performed using a C1000 Touch Thermal Cycler (BioRad) with the following cycling parameters: enzyme activation at 95°C for 5 minutes, denaturation at 95°C for 30 seconds, annealing/elongation at 57°C for 1 minute for 40 cycles, signal stabilization at 4°C for 5 minutes and 95°C for 5 minutes, and hold at 4°C. Following amplification, the droplets were transferred to a QX100 droplet reader and analyzed using Quantasoft (BioRad) software.

### Prion infections, clinical scoring and mouse dissections

RML-5 prions were prepared as previously described [79]. 10% RML-5 brain homogenates were diluted 1:10 in 1X PBS supplemented with 100 units/mL of Penicillin and 100 μg/mL of Streptomycin (Gibco) immediately before inoculation. Prion titers were determined using the relationship:

y=11.45‐0.088x,

where y is lethal dose 50 (LD_50_) units and x is the incubation time in days to terminal disease [78]. We injected 100 μL of inoculum containing 3.3 x 10^6^ LD_50_ units, in the left or right bottom quadrant of the intraperitoneal cavity, or 30 μL (10^6^ LD_50_ units) injected intracerebrally 3 mm deep through the coronal suture 3–5 mm lateral of the sagittal suture, with a 29-gauge insulin syringe (BD). Mice were monitored daily and sacrificed at the onset of terminal disease or specified time points. We scored mice for clinical prion disease as previously described [103]. Briefly, we employed a scoring system to assess the severity of disease, including: tail rigidity (0–2), akinesia (0–4), ataxia (0–4), tremors (0–4), and weight loss (0–4). Mice scored above 10 or 4 in any single category were considered terminally ill and immediately euthanized via CO_2_ inhalation, replacing 20% of air per minute to effect. Brain, spleen, and sera were collected from each mouse at time of euthanasia. Half of the brain and spleen were frozen at -20°C, and the other halves were fixed in 4% paraformaldehyde.

### Behavioral testing

Behavioral tests were started approximately midway through disease course. For burrowing, mice were given approximately 100 grams of food stuffed into a 6-inch plastic PVC pipe. Mice were allowed to burrow out the food for 30 minutes. Rate of burrowing was calculated by the number of grams of food removed divided by total time burrowed. For nesting, mice were given a small cotton nestlet and allowed to build a nest overnight. Mice were scored on a scale from 0–4, with 0 being no nest and 4 being a normally built nest. Average nesting scores were calculated for each treatment group.

### Immunohistochemistry

Brains from prion-infected mice from the 1^st^ LSPCs terminal study were sent to Colorado State University’s Veterinary Diagnostic Laboratory (VDL) for paraffin embedding, sectioning and GFAP staining. Unprocessed sections were stained for PrP^Res^ using the following protocol. Slides were incubated at 53°C for 30 minutes before being immersed in xylene twice for 10 minutes. The slides were then rehydrated through an ethanol gradient consisting of 100%, 95% and 70% concentrations for 5 minutes each and then immersed in 88% formic acid for 10 minutes. After washing the slides in running water for 10 minutes, the slides were processed through antigen retrieval while in citrate buffer, pH of 7.4. The slides were allowed to cool before being washed twice in a 0.1% PBS-Triton buffer for 5 minutes on a rocker. Slides were immersed in a 3% hydrogen peroxide preparation in methanol for 30 minutes to extinguish exogenous peroxidase activity of the tissues before undergoing another wash cycle. Tissues were then encircled with a hydrophobic barrier and allowed to incubate with Superblock (Pierce) for 30 minutes. The excess block was tapped off each slide and the slides were incubated overnight in 4°C with D18 anti-PrP monoclonal antibody at a 1:1000 dilution. Slides were washed and incubated with a biotinylated anti-human Ig (1:1000) for 1 hour at room temperature. They were then washed and incubated with streptavidin solution for 30 minutes at room temperature. After 3 wash cycles, the slides were incubated with diaminobenzidine reagent for 5 minutes to develop the staining. Slides were then washed and counterstained in hematoxylin for 5 minutes, and then immersed in water for 10 minutes to deactivate the hematoxylin. Slides were dehydrated through the alcohol gradient and xylene before being mounted with a coverslip. Slides were visualized using a BX-60 microscope and pictures recorded using a cooled charge-coupled diode camera (Olympus). We quantified PrP^Res^ and GFAP signal intensities using the CMYK color model and Graphic Converter 10 (Lemke Software) as previously described [79,104,105]. We quantified vacuolation by manual counting in at least three non-consecutive sections per mouse and at least two mice per group.

### ELISA for RVG-9r-specific IgG

Serum samples from terminally ill mice were collected by heart stick after euthanasia. Samples were stored at -20°C until assay was performed. 1 μg of RVG-9r was coated into 96-well ELISA plates (Nunc) using carbonate/bicarbonate buffer (Sigma). The plates incubated overnight at 4°C, then washed 2X with ELISA wash buffer (1X PBS + 0.05% Tween). All wells were blocked with SuperBlock (ThermoFisher) at room temperature for 2 hours. Plates were washed 2X with ELISA wash buffer. The following serum dilutions from LSPCs-treated mice were dispensed onto the plate: 1:50, 1:100, 1:250, 1:500, 1:1000, and 1:2000. The serum was incubated overnight at 4°C. All wells were washed 4X with ELISA wash buffer. A 1:5000 dilution of an anti-mouse IgG horseradish peroxidase secondary antibody (Cell signaling) in SuperBlock was added to each well and incubated at room temperature for 2 hours. All wells were washed again with ELISA wash buffer. TMB substrate (ThermoFisher) was added to each well and allowed to incubate until a deep blue color change developed. To stop the reaction, a stop solution (0.5 M H_2_SO_4_ in 1X PBS) was added to each well. Photometric analysis was performed at 450 nm using a Multiskan Spectrum plate reader (ThermoFisher).

### PK digestion and western blots

Proteinase K (Roche) was added to western blot samples at a 1:10 dilution for a final concentration of 50 μg/mL. The samples were incubated at 37°C for 30 minutes with a 10-minute deactivation step at 95°C. Proteins were electrophoretically separated using 12% sodium dodecyl sulfate polyacrylamide gels (Invitrogen). Proteins were then transferred to a polyvinylidene difluoride membrane (Millipore). Membranes were blocked using 5% non-fat dry milk for 1 hour, washed 2X for 10 minutes each using 1X PBS with 0.2% Tween, then incubated with horseradish peroxidase-conjugated BAR-224 (SPI Bio) anti-PrP^C^ antibody diluted 1:20,000 overnight at 4°C. Membranes were washed again 6X for 10 minutes each and incubated with enhanced chemiluminescent substrate (Millipore) for 5 minutes. Membranes were photographed using an ImageQuant LAS 4000 (GE).

### Statistical analysis

We performed statistical analyses using GraphPad Prism and report specific tests, parameters, results and significance values for each experiment for which the test was used.

## Supporting information

S1 Fig Additional control LSPCs demonstrate PrP siRNA LSPC specificity. Naïve FVB mice were injected intravenously with PBS, PrP siRNA LSPCs, scrambled PrP siRNA LSPCs, irrelevant siRNA LSPCs or RVM-9r LSPCs. Protein and mRNA expression were analyzed four days after treatment by flow cytometry and ddPCR, respectively. Only PrP siRNA L SPCs significantly reduced PrP mRNA (A) and protein (B) in the brain. Scrambled PrP siRNA LSPCs significantly increased PrP mRNA in both brains and kidneys, but reduced PrP protein expression in kidneys. All other controls did not significantly affect PrP mRNA and protein expression. Error bars indicate 95% CI of the mean. * p<0.05, ** p<0.01, *** p<0.001, **** p<0.0001. One-way ANOVA with Dunnett’s multiple comparisons.
(TIFF)

S2 Fig **We compared (A) IN versus (B) IV delivery of LSPCs on survival of prion infected mice.** (A) IN responders survived significantly longer (dotted red line, n = 3, median survival, 238 DPI) than IN non-responders (dotted black line, n = 2, 217 and 227 DPI, p < 0.05). (B). IV responders similarly lived significantly longer (dotted red line, n = 3, median survival 232 DPI) than IV non-responders (dotted black line, n = 9, median survival 216 DPI).
(TIFF)

S3 Fig IgG levels against RVG-9r in infected TgA20 mice treated 1-3X with LSPCs. We detected no significant RVG-9r titers in any group, the data from which we compared to data from uninfected, treated wild type mice reported in Fig 6A.
(TIFF)

S1 Table Treatment groups. Breakdown of control and treated groups in the 1^st^ (Panels A and B) and 2^nd^ (Panels C and D) LSPC treatment studies, along with days post infection each LSPC treatment was given.
(TIFF)

S2 Table Treatment regimen. DPI of LSPC treatment and euthanasia of early time point mice treated with LSPCs to assess minimal LSPCs treatments required for an immune response.
(TIFF)
