# Supplementary figures and images for: PrPC knockdown by liposome-siRNA-peptide complexes (LSPCs) prolongs survival and normal behavior of prion-infected mice immunotolerant to treatment
Source: PLoS One. 2019 Jul 22;14(7):e0219995. doi: 10.1371/journal.pone.0219995 (PMC6645518; doi:10.1371/journal.pone.0219995)

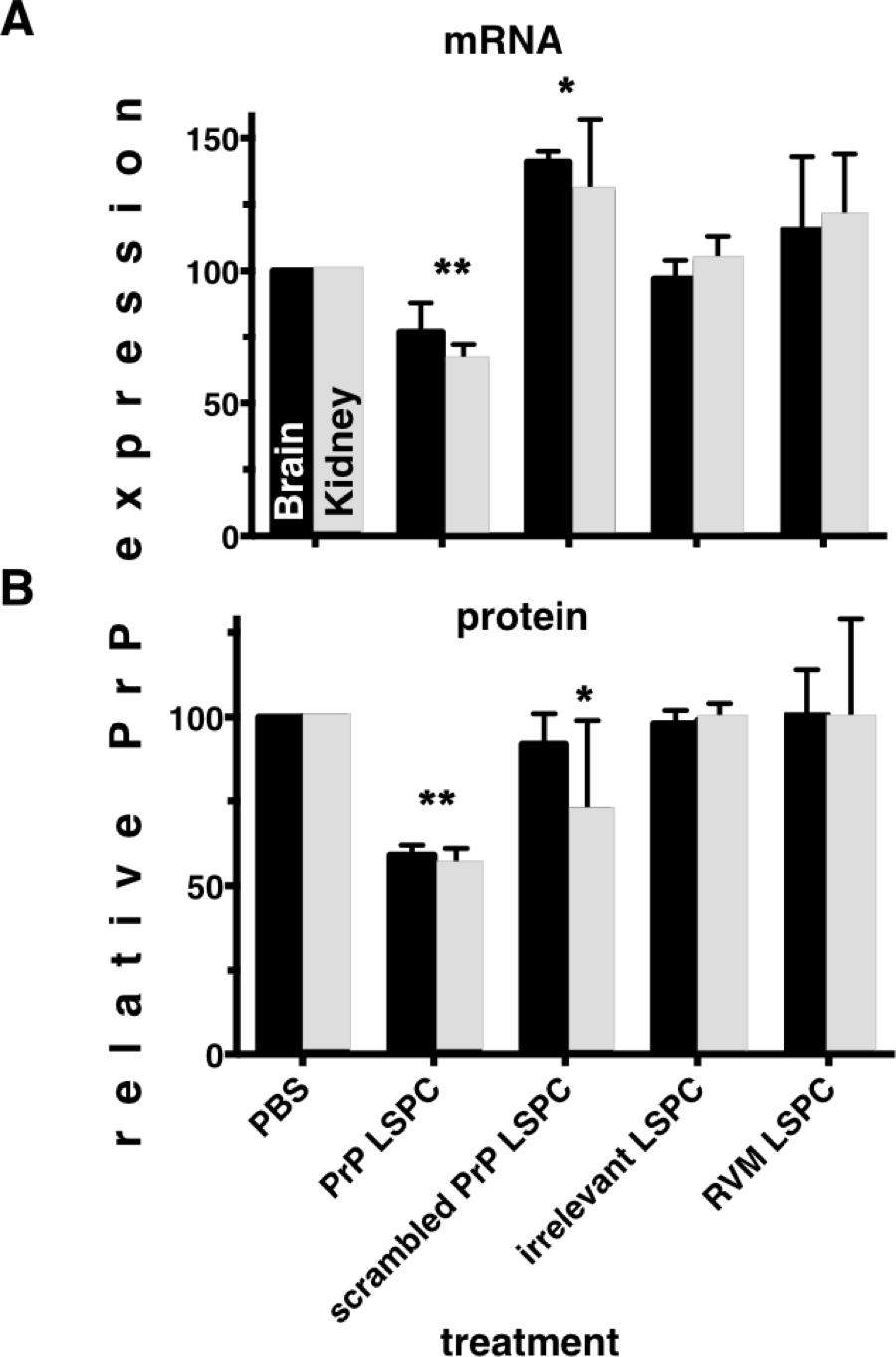

Supplement: S1 Fig — Naïve FVB mice were injected intravenously with PBS, PrP siRNA LSPCs, scrambled PrP siRNA LSPCs, irrelevant siRNA LSPCs or RVM-9r LSPCs. Protein and mRNA expression were analyzed four days after treatment by flow cytometry and ddPCR, respectively. Only PrP siRNA L SPCs significantly reduced PrP mRNA (A) and protein (B) in the brain. Scrambled PrP siRNA LSPCs significantly increased PrP mRNA in both brains and kidneys, but reduced PrP protein expression in kidneys. All other controls did not significantly affect PrP mRNA and protein expression. Error bars indicate 95% CI of the mean. * p<0.05, ** p<0.01, *** p<0.001, **** p<0.0001. One-way ANOVA with Dunnett’s multiple comparisons. (TIFF) [file pone.0219995.s001.tiff]

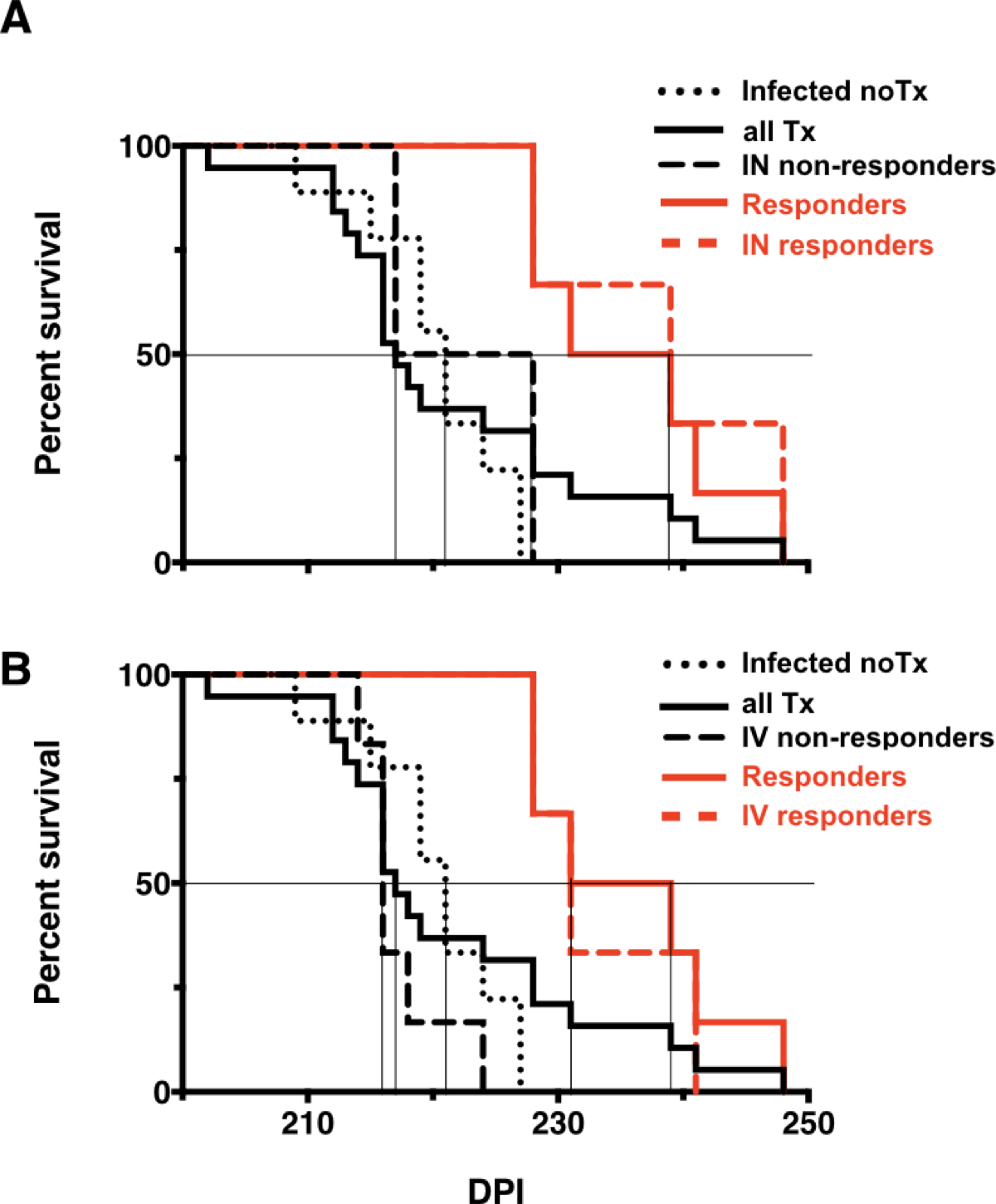

Supplement: S2 Fig — We compared (A) IN versus (B) IV delivery of LSPCs on survival of prion infected mice. (A) IN responders survived significantly longer (dotted red line, n = 3, median survival, 238 DPI) than IN non-responders (dotted black line, n = 2, 217 and 227 DPI, p < 0.05). (B). IV responders similarly lived significantly longer (dotted red line, n = 3, median survival 232 DPI) than IV non-responders (dotted black line, n = 9, median survival 216 DPI). (TIFF) [file pone.0219995.s002.tiff]

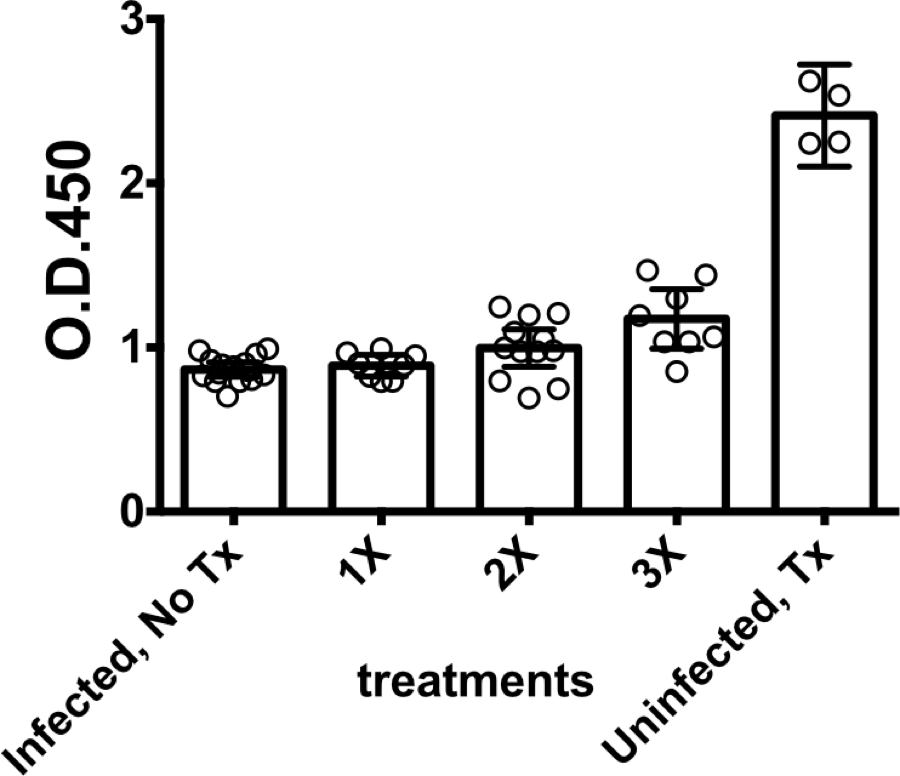

Supplement: S3 Fig — We detected no significant RVG-9r titers in any group, the data from which we compared to data from uninfected, treated wild type mice reported in Fig 6A. (TIFF) [file pone.0219995.s003.tiff]

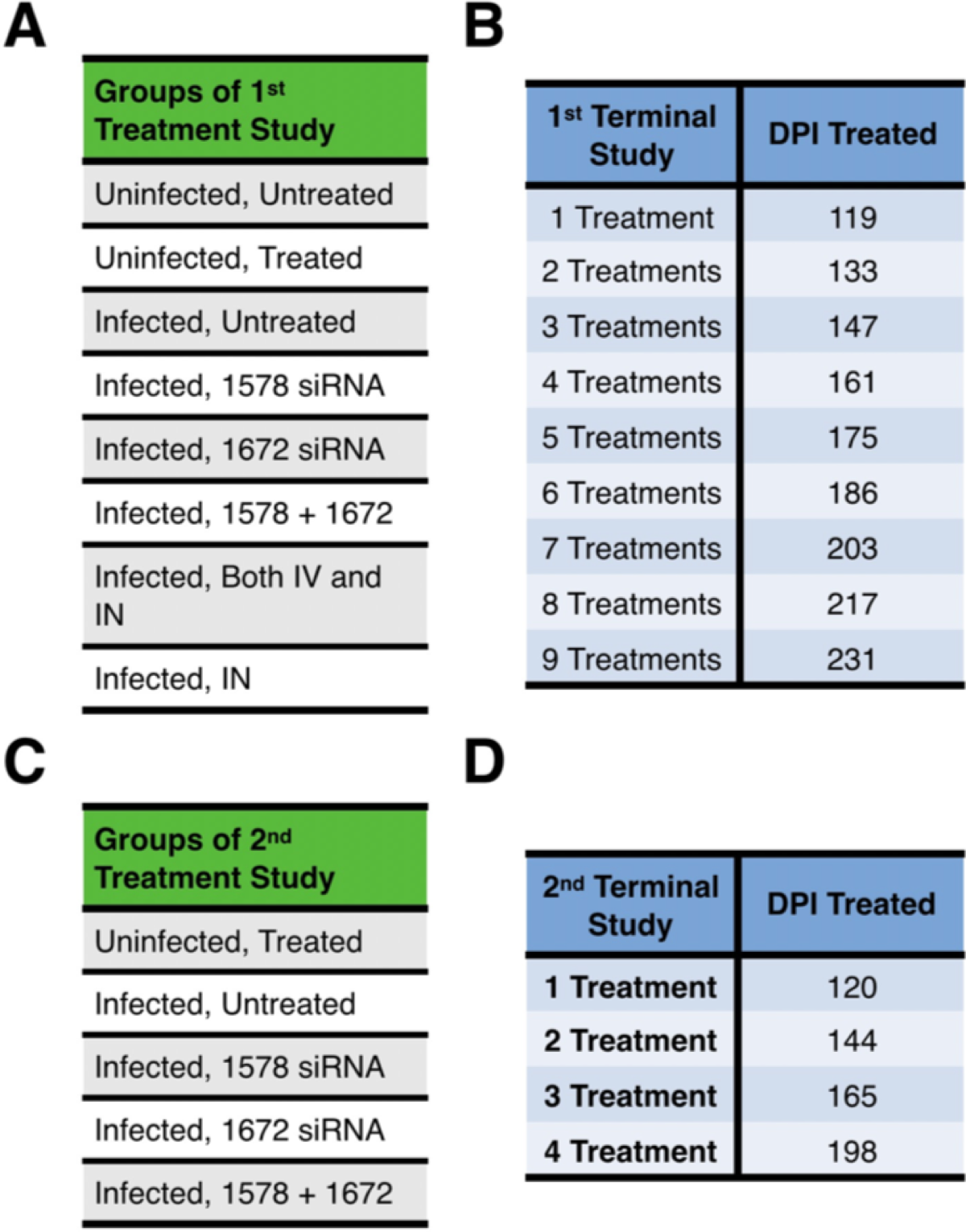

Supplement: S1 Table — Breakdown of control and treated groups in the 1st (Panels A and B) and 2nd (Panels C and D) LSPC treatment studies, along with days post infection each LSPC treatment was given. (TIFF) [file pone.0219995.s004.tiff]

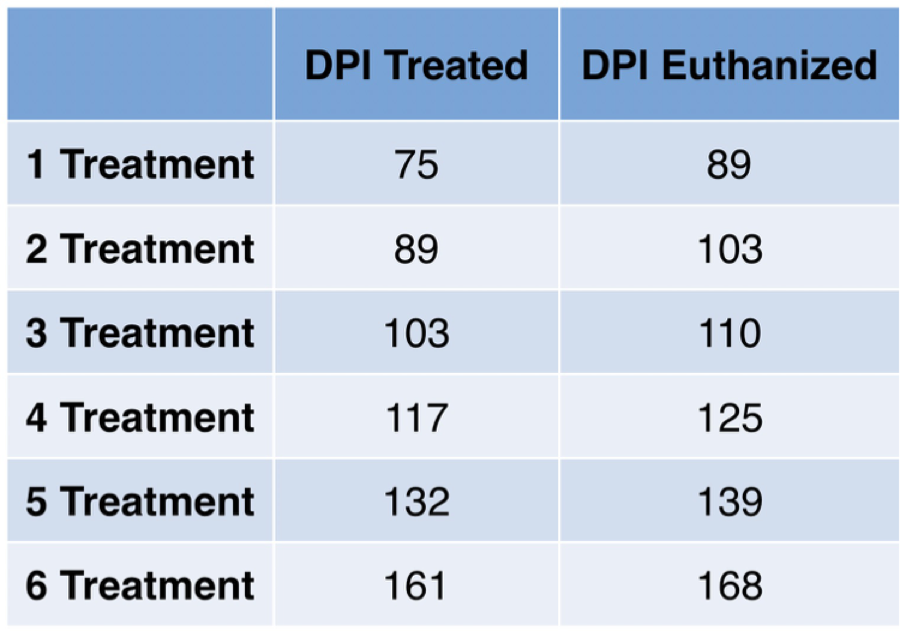

Supplement: S2 Table — DPI of LSPC treatment and euthanasia of early time point mice treated with LSPCs to assess minimal LSPCs treatments required for an immune response. (TIFF) [file pone.0219995.s005.tiff]
